# Topical Nanoemulsions as Delivery Systems for Green Extracts of *Pterocaulon balansae* Aiming at the Treatment of Sporotrichosis

**DOI:** 10.3390/pharmaceutics16040492

**Published:** 2024-04-02

**Authors:** Bruna Medeiros-Neves, Daiane Heidrich, Roselena Silvestri Schuh, Nathalya Tesch Brazil, Flávia Nathiely Silveira Fachel, Eduardo Cassel, Rubem Mário Figueiró Vargas, Maria Lúcia Scroferneker, Gilsane Lino von Poser, Letícia Scherer Koester, Helder Ferreira Teixeira

**Affiliations:** 1 Programa de Pós-Graduação em Ciências Farmacêuticas, Faculdade de Farmácia, Universidade Federal do Rio Grande do Sul, Av. Ipiranga 2752, Porto Alegre 90610-000, RS, Brazil; bruna.medeiros@ufrgs.br (B.M.-N.); roselena.schuh@ufrgs.br (R.S.S.); nathalya.brazil@ufrgs.br (N.T.B.); flavia.fachel@ufrgs.br (F.N.S.F.); gilsane@farmacia.ufrgs.br (G.L.v.P.); leticia.koester@ufrgs.br (L.S.K.); 2 Departamento de Microbiologia, ICBS, Universidade Federal do Rio Grande do Sul, Rua Sarmento Leite, Porto Alegre 90050-170, RS, Brazil; daiane.heidrich@univates.br (D.H.); scrofern@ufrgs.br (M.L.S.); 3 Faculdade de Engenharia, Departamento de Engenharia Química, Pontifícia Universidade Católica do Rio Grande do Sul, Av. Ipiranga, 6681-Prédio 30-Sala 277, Porto Alegre 90619-900, RS, Brazil; cassel@pucrs.br (E.C.); rvargas@pucrs.br (R.M.F.V.)

**Keywords:** coumarin, *Sporotrix schenckii*, permeation/retention assay, nanoemulsion, high-pressure homogenization

## Abstract

Coumarins are benzopyrones found in several plant genera, including *Pterocaulon* (Asteraceae). These compounds represent an important source of new treatments, especially as antimicrobial and antifungal agents. In this study, two coumarin-rich extracts from *Pterocaulon balansae* using green technologies were obtained through aqueous maceration (AE) and supercritical fluid extraction (SFE). Such extracts were incorporated into nanoemulsions (NAE and NSFE) composed of a medium-chain triglyceride oil core stabilized by phospholipids. The nanoemulsions exhibited droplet sizes between 127 and 162 nm, pH above 5.0, and viscosity of approximately 1.0 cP, properties compatible with the topical route. The coumarins permeation/retention from formulations through ear porcine skin using Franz-type diffusion cells were evaluated. Whatever the extract, coumarins were distributed in skin layers, especially in the dermis in both intact and impaired (tape stripping) skin. In addition, a significant increase in coumarins that reached up to the receptor fluid was observed for impaired skin, with increases of approximately threefold for NAE and fourfold for NSFE. Finally, antifungal activity of nanoemulsions was evaluated according to minimum inhibitory concentrations, and the values were 250 µg/mL for all strains tested. The overall results demonstrated the feasibility of incorporating *P. balansae* extracts into nanoemulsions and showed a potential alternative for the treatment of sporotrichosis.

## 1. Introduction

Coumarins are secondary metabolites of benzopyrones found in several plant genera. According to various studies, these compounds and their derivatives represent an important source of new treatments, especially as antioxidants, antimicrobials, antineoplastics, and anti-inflammatory agents [1,2,3,4,5,6]. Coumarins are the major compounds present in *Pterocaulon* Ell. species, and recent reports have described the antifungal activity of their extracts [7].

Stopiglia et al. assessed the in vitro antifungal potential of five plant species from the *Pterocaulon* genus, namely *P. balansae*, *P. cordobense*, *P. lanatum*, *P. lorentzii*, and *P. polystachyum*, against 24 strains of *Sporothrix schenckii* [8]. Their investigation revealed that methanolic extracts, when applied at a concentration of 10%, exhibited both inhibitory and fungicidal effects [8]. *S. schenckii* is the causative agent responsible for sporotrichosis, a prevalent subcutaneous mycosis affecting both humans and animals. This disease is globally distributed, with a higher incidence in tropical and subtropical regions, particularly in Japan, India, South Africa, Mexico, Peru, Uruguay, and Brazil. In South America, the estimated annual incidence ranges from 48 to 60 cases per 100,000 people. The fungus typically infiltrates the skin, where it can persist within subcutaneous tissue or migrate along adjacent lymphatic vessels. Clinical manifestations of sporotrichosis include the development of skin lesions in the form of papules or pustules, which subsequently evolve into subcutaneous nodules [9,10,11]. The primary treatment approach involves a combination of potassium iodide and itraconazole, which is generally effective. However, the prolonged duration of therapy and the occurrence of toxic side effects necessitate the exploration of alternative treatment options for severe infections [3].

Most biological studies have been performed with methanolic or hexanic extracts of *P. balansae* [7]. Nevertheless, green technologies are preferred for industrial application. This study explores the benefits of using green technology, known for its environmentally friendly processing methods and, in certain instances, as a viable substitute for conventional organic solvent-based extraction of natural products [12,13].

Traditional delivery systems, such as creams, lotions, and gels, are hindered by challenges like poor drug penetration, reduced efficacy, and sensitization reactions when delivering antifungal agents. Nanoformulations, especially lipid-based nanocarriers, have gained prominence in addressing these issues for topical treatment of fungal infections [14]. The use of nanoemulsions as systems for incorporating plant extracts to enable their topical administration has been widely described as improving the characteristics of the compounds, such as by promoting greater solubility, stability, and penetration into the skin layers, of the herbal bioactives through encapsulation [15]. Even so, the chemical profile of the extract may impact the association of the coumarins to the nanoformulation and the potentiation of the biological activity.

Following up on these results, the objective of this study was to assess the cutaneous penetration and retention of coumarins from nanoemulsions that contain two different extracts from *P. balansae* (aqueous and supercritical fluid) as well as to assess their antifungal activity against *S. schenckii* strains, which is yet unexplored.

## 2. Materials and Methods

### 2.1. Plant Material

The aerial parts of *P. balansae* Chodat. (Asteraceae) were gathered in Canoas, RS, Brazil, and they were subsequently authenticated by the botanist Sérgio A. L. Bordignon, affiliated with Centro Universitário La Salle, Canoas, Unilasalle, Brazil. The corresponding voucher specimens were archived in the herbarium of Universidade Federal do Rio Grande do Sul (ICN 157762). The acquisition of these plant specimens was legally sanctioned and authorized by the Ministério do Meio Ambiente (Nº 38017-1, Sistema de Autorização e Informação em Biodiversidade).

### 2.2. Materials

The solvents acetonitrile and formic acid were obtained from Tedia (HPLC grade, Radnor, PA, USA), and ultrapure water was obtained from the Milli-Q^®^ Plus apparatus by Millipore (Billerica, MA, USA). Methanol, ethanol, isopropanol, polysorbate 80 (Tween^®^ 80), dimethyl sulfoxide (DMSO), and sodium hydroxide were obtained from Vetec (Duque de Caxias, Brazil), and medium chain triglycerides (MCT) and egg lecithin (Lipoid^®^ E80) were purchased from Lipoid GmbH (Ludwigshafen am Rhein, Germany). Monobasic potassium phosphate was obtained from Dinâmica (São Paulo, Brazil), and NBD-PE [N-(7-nitrobenz-2-oxa-1,3-diazol-4-yl)-1,2-dihexadecanoyl-sn-glycero-3-phosphoethanolamine, triethylammonium salt] fluorescent-labelled phospholipid was purchased from Thermo Fisher Scientific (Waltham, MA, USA). Roswell Park Memorial Institute 1640 broth medium (RPMI-1640), 3(N-morpholino) propane sulfonic acid (MOPS), 3-(4,5-dimethylthiazol-2-yl)-2,5-diphenyltetrazolium bromide (MTT), and itraconazole were obtained from Sigma-Aldrich (St. Louis, MA, USA), and potato dextrose agar (PDA) was purchased from Acumedia (San Bernardino, CA, USA). Tissue-Tek^®^ O.C.T.™ was purchased from Sakura Finetechnical Co. (Tokyo, Japan). Log P (calculated log P) values of the coumarins were assigned using SwissADME http://www.swissadme.ch/ (accessed on 10 March 2023).

### 2.3. Strains

The clinical isolates used in this study were sourced from the Mycology Collection at the Pathogenic Fungi Laboratory within the Department of Microbiology at the Institute of Basic Health Sciences, Universidade Federal do Rio Grande do Sul, Brazil. Specifically, four clinical isolates of *S. schenkii* (identified as Santa Casa 1; MLS; 31 UCS; STT) were obtained from this repository. Additionally, one isolate was obtained from the American Type Culture Collection (ATCC 201679; Rockville, MD, USA).

### 2.4. Pterocaulon Balansae Extraction

The extracts were obtained from the aerial parts of *P. balansae*. Aqueous extract (AE) was obtained through a previously described methodology [16]. Briefly, the dried aerial parts were extracted with water 2% (*w*/*v*) at 60 °C in a water bath (Dist DI920) for approximately 4 h. The supercritical fluid extract (SFE) was also obtained according to a previously described method [17] where extractions were carried out in the pilot unit of supercritical extraction and extracted at a constant temperature of 40 °C and 120 bar. The solvent flow rate used in the extractions was 6.7 × 10^−4^ kg s^−1^, and the extraction time was set at 2 h. The samples were stored for subsequent studies.

### 2.5. Preparation of Nanoemulsions

Three distinct formulations were prepared: (i) blank nanoemulsion (NB); (ii) aqueous extract-loaded nanoemulsion (NAE); and (iii) supercritical fluid extract-loaded nanoemulsion (NSFE). The nanoemulsions were prepared at the concentration of 1.6 mg/mL of total coumarins and obtained through high-pressure homogenization (HPH). Briefly, the oily phase (16% *w*/*w* MCT, 4% *w*/*w* egg lecithin) and the aqueous phase (1% *w*/*w* Tween 80^®^ and 100% water) were mixed under magnetic stirring (15 min at room temperature) to form a coarse emulsion. For NAE, the AE was solubilized in the aqueous phase at room temperature due to the affinity, and, to prepare the NSFE, the SFE was dispersed in the oil phase at 30 °C. The initial coarse emulsions were blended for 2 min at 9500 rpm employing an IKA^®^ Ultra-Turrax T8 mixer (IKA^®^ Works Inc., Wilmington, NC, USA) to generate preliminary pre-emulsions. These pre-emulsions underwent a subsequent step using high-pressure homogenization (EmulsiFlex-C3^®^, Avestin, Saint-Jérôme, QC, Canada) operating at 750 bar (equivalent to 10,000 psi) for 10 cycles, aiming to systematically reduce droplet sizes and form the desired nanoemulsions. The specific compositions of the final formulations can be found in Table 1.

### 2.6. Physicochemical Properties of Nanoemulsions

#### 2.6.1. Droplet Size, Polydispersity Index (PDI), and ζ-Potential

The samples were diluted with water previously filtered in 0.22 μm of membrane for the assessment of droplet size and the PDI using photon correlation spectroscopy at a temperature of 25 °C. Additionally, the ζ-potential was determined through electrophoretic mobility. These measurements were conducted utilizing a Zetasizer Nano-ZS90^®^ (Malvern Instruments, Malvern, UK). The outcomes are presented as the average values derived from three separate determinations.

#### 2.6.2. pH and Viscosity Measurements

The pH of the nanoemulsions was measured directly using a previously calibrated potentiometer (Model pH UltraBasic, Denver Instruments, Arvada, CO, USA). The results were represented as the average of three distinct assessments. The viscosity of the nanoemulsions was measured through capillary viscometry in an Ostwald viscometer at 23 ± 0.1 °C using a Number 2 capillary and 2 mL aliquots of each nanoemulsion. The relative viscosity (cP) was calculated by considering the flow time through the capillary and the density of the formulations. Each of these measurements was conducted three times for accuracy.

#### 2.6.3. NBD-PE-Labelled Nanoemulsions

The NBD-PE fluorescent-labelled phospholipid (Thermo Fisher Scientific, USA) was dissolved in the oil phase of the nanoemulsions in a 1% formulation proportion. Preparation of the formulation was carried out following the instructions outlined in Section 2.5.

### 2.7. Permeation/Retention Assay in Intact and Impaired Porcine Skin

Porcine ears were acquired from a nearby slaughterhouse. The procedure for obtaining ears for the experiment is described in Argenta et al. (2014) [18]. The permeation/retention assay was conducted using Franz-type diffusion cells with porcine ear skin as the membrane. The entire experiment was maintained at a controlled temperature of 32 ± 1.0 °C, with continuous stirring at 480 rpm, according to OECD 428 [19]. The skin sections were hydrated with pH 7.4 PBS for 15 min at room temperature before being placed in the cell. The circular sections of porcine skin were deposited on top of the cells, between the donor and receptor compartments of the Franz cell, on a surface area of 1.77 cm^2^. The receptor compartment was supplied with a PBS:ethanol (60:40) mixture. A volume corresponding to 550 µL of NAE or NSFE formulation was then applied to the donor compartment. The mixture of solvents (PBS:ethanol) employed and the volume of formulation were added to attend the sink condition. Simulating a daily application of the formulation, after 8 h, a portion of the receptor fluid was collected, and the skin was extracted from the cell. Circular segments were cleaned, and the epidermis was isolated from the dermis and subsequently diced into small pieces. The coumarins were extracted with methanol using an ultrasonic bath for 45 min. Once the spore infections affected the deeper layers of the skin, the experiments were also performed in damaged tissues by analyzing the total coumarin permeation/retention extent in this condition. The previous consolidated literature has reported the use of the tape stripping technique to injure the skin by removing the stratum corneum [20]. Therefore, for evaluation of impaired skin, tape stripping was performed using 20 tapes (Scotch 750 tape, 3 M) before the experiment. All of the samples were analyzed through UFLC [16], and the results are expressed as µg total coumarins per skin area.

### 2.8. Histological and Confocal Microscopy

Following the permeation assay, the skin samples were prepared in accordance with the methodology reported by Argenta et al. (2014) for subsequent histological and confocal microscopy examinations [18]. For histological analysis, the specimens were subjected to staining with haematoxylin and eosin (H&E) and subsequently photographed using optical microscopy at a 100× magnification. In the case of confocal fluorescence experiments, the incorporation of a fluorescent dye (NBD-PE) occurred during the initial preparation of the nanoemulsions through HPH. Each fluorescent nanoemulsion (approximately 550 μL) was placed in the donor compartment, and the permeation/retention study was executed under identical experimental conditions as those detailed in Section 2.7 above. Following intervals of 1 and 8 h, the skin samples underwent cleaning and were then mounted with Tissue-Tek^®^ O.C.T.™ on a metal sample holder before being frozen at −20 °C. Subsequently, vertical slices of these skin samples, with a thickness of 30 μm, were acquired using a cryostat (CM 1850; Leica Microsystems, Wetzlar, Germany), and these slices were subsequently analyzed using a fluorescence microscope (Olympus BX51TF, Tokyo, Japan) at both 100× and 400× magnifications.

### 2.9. Ultra Fast Liquid Chromatography Analyses

The total coumarin (TC) contents of extracts (AE, SFE), nanoemulsions (NAE, NSFE), porcine skin (epidermis and dermis), and receptor fluid after permeation studies were determined using a previously validated UFLC method [16]. Adequate aliquots of the samples were diluted in CH_3_CN:H_2_O (1:1 *v*/*v*), filtered, and analyzed in UFLC.

### 2.10. Antifungal Activity

In accordance with the guidelines recommended by the Clinical and Laboratory Standards Institute (CLSI) for filamentous fungi, specifically M38-A2 [21], we conducted antifungal susceptibility assays using the broth microdilution method. The fungal strains were initially subcultured on potato dextrose agar at a temperature of 35 °C for a duration of 7 days. Following this incubation period, the agar surface was gently scraped using a sterile bent glass rod after being thoroughly soaked with sterile saline solution. Standardized suspensions were prepared, and their optical densities were adjusted to fall within the range of absorbance between 0.09 and 0.13 using UV-visible spectrophotometry (Spectrum Instruments Co., Shanghai, China). These adjusted suspensions were further diluted in RPMI-MOPS (1:50) to achieve a final inoculum concentration of 10^4^ CFU/mL. Subsequently, 100 µL of the fungal suspensions was introduced into each microdilution well, which also contained 100 µL of the samples (AE, SFE, NAE, and NSFE). The final concentrations of these samples spanned from 3.906 to 1000 µg/mL. This same procedure was followed for NB, the positive control (itraconazole), the growth control (untreated microorganisms), and the sterility control (absence of microorganisms). The stock solution of itraconazole (1600 µg/mL) was prepared in DMSO, while other solutions were appropriately diluted in RPMI-MOPS to attain final concentrations ranging from 0.0313 to 16 µg/mL. Subsequently, the prepared plates were incubated at 35 °C for a period of 4 days, followed by a comprehensive analysis of the results.

#### 2.10.1. Minimum Inhibitory Concentration (MIC)

Determination of the MIC was performed visually through comparison with the growth control. The MIC was characterized as the lowest concentration of the treatments (including extracts and nanoemulsions) capable of entirely suppressing fungal growth. All of the experiments were carried out in triplicate to ensure accuracy and consistency.

#### 2.10.2. Hyphal Damage Assay with MTT

To evaluate the hyphal damage caused by the treatments, a colorimetric assay using MTT was performed [22]. After determination of the MIC, the same plates were used for the MTT assay. Initially, 150 µL of the supernatant was discarded and 150 µL of MTT was added (0.005% *w*/*v*) to the wells, which were incubated for 3 h at 37 °C. Following the removal of the MTT suspension, the MTT formazan crystals were extracted from the hyphae using 150 µL of isopropanol. The mixture was homogenized, and 100 µL of the resulting supernatant was subsequently transferred to a flat-bottom 96-well plate. The absorbance (A) was measured using a multiscan-enzyme-linked immunosorbent assay reader (Titertek MCC/340; Labsystems, Helsinki, Finland) at a dual wavelength of 570 and 690 nm. The percentage of hyphal damage was calculated using the following formula:

(1) $$Cell\;Demage\;/\%=1-\frac{A570-A690\;with\;drugs}{A570-A690\;without\;drugs}\times 100$$

Non-specific absorption was accounted for by subtracting the absorbance at 690 nm from the absorbance at 570 nm.

### 2.11. Statistical Analyses

Significant statistical differences were determined through analysis of variance with Tukey’s post hoc test (* *p* < 0.05, ** *p* < 0.001, *** *p* < 0.0001). Parameters were analyzed using the software GraphPad Prism (version 6.01, GrapPad software Inc., San Diego, CA, USA).

## 3. Results and Discussion

### 3.1. Preparation and Characterization of Nanoemulsions

**Figure 1:**
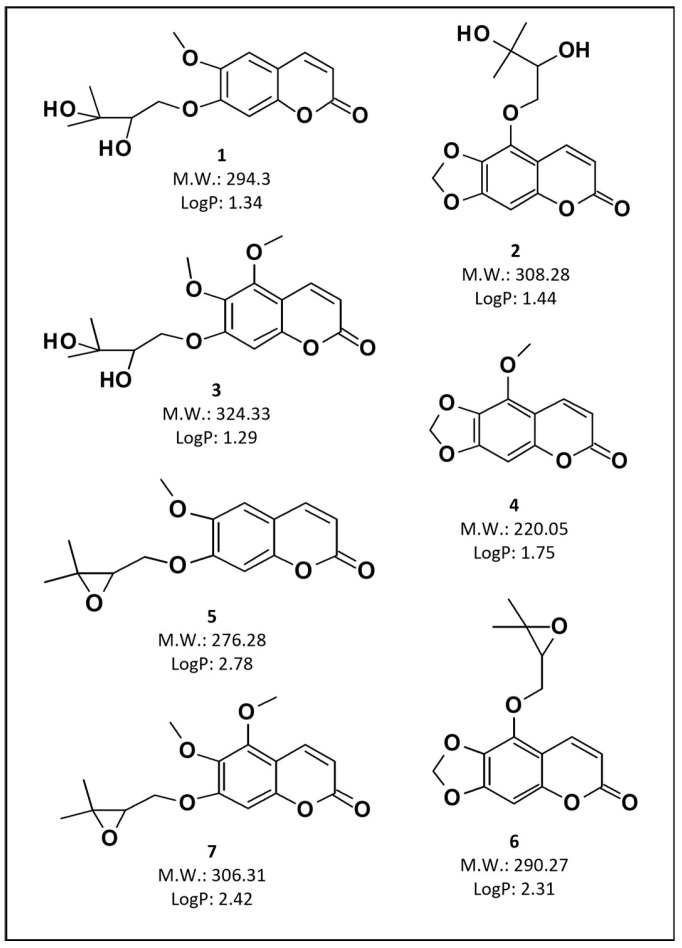
Structure, molecular weight, and LogP of the coumarins described in *P. balansae* extracts. (**1**) 7-(2′,3′-dihydroxy-3′-methylbutyloxy)-6-methoxycoumarin, (**2**) 5-(2′,3′-dihydroxy-3′-methylbutyloxy)-6,7-methylenedioxycoumarin, (**3**) 5,6-dimethoxy-7-(3′-methyl-2′,3′-dihydroxybutyloxy)coumarin, (**4**) 5-methoxy-6,7-methylenedioxycoumarin, (**5**) 7-(2′,3′-epoxy-3′-methyl-3′-butyloxy)-6-methoxycoumarin, (**6**) 5-(2′,3′-epoxy-3′-methylbutyloxy)-6,7-methylenedioxycoumarin, and (**7**) 5,6-dimethoxy-7-(2′,3′-epoxy-3′-methylbutyloxy)coumarin.

**Figure 2:**
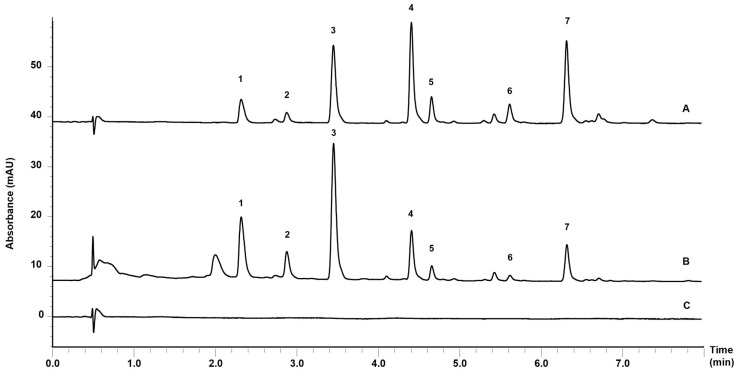
Chromatographic profile of (**A**) supercritical fluid extract-loaded nanoemulsion (NSFE); (**B**) aqueous extract-loaded nanoemulsion (NAE); and (**C**) blank nanoemulsions (NB). The numbering of the chromatogram peaks corresponds to the structures and chemical names presented in Figure 1.

In the present study, two nanoemulsions loaded with different *P. balansae* extracts were prepared to evaluate their permeation/retention in intact and impaired porcine skin, as well as to investigate their antifungal activity against *S. schenkii*. Initially, the AE and SFE were obtained according to previous studies [16,17]. The coumarins hitherto described for this species are shown in Figure 1. According to the chromatographic profile of the NAE and NSFE formulations (Figure 2), it is possible to observe quantitative differences in the content of these coumarins.

In the chromatogram of the NAE, a greater amount of coumarins 1–3 was observed. These compounds, due to their chemical properties, exhibit a more hydrophilic character, confirmed by the log *p* values (1.29–1.44). Therefore, they are obtained in larger quantities when water is the solvent. In the NSFE, on the contrary, the opposite behavior is observed because this process enables the extraction of lipophilic coumarins, as can be observed in Figure 2, where there is a predominance of coumarins 4–7 in the formulation.

The next step involved the incorporation of these extracts in nanoemulsions. Three formulations were prepared (NB, NAE, and NSFE) through HPH. HPH is a prevalent technique in the pharmaceutical industry employed to achieve size reduction by compelling a coarse emulsion through a homogenizing valve at elevated pressures. This process results in the deformation and reduction of droplet size [23].

Nanoemulsions offer advantages compared to conventional microemulsions, particularly regarding surfactant concentration. While microemulsions typically require a surfactant concentration of 20% or higher, nanoemulsions can be prepared with lower concentrations ranging from 3% to 10%. Additionally, nanoscale particles enable a more uniform distribution of formulations on the skin, high stability, and the ability to improve the solubility of compounds, in turn improving bioavailability. Therefore, nanoemulsions are a suitable option for the efficient delivery of active ingredients through the skin [14,24]. Table 2 presents the physicochemical properties of the nanoemulsions. The total coumarin content in the formulations was higher than 90%, demonstrating the efficient association of coumarins to these systems, which were incorporated in the oil nucleus and/or adsorbed on the interface of lipid droplets. In other words, we added an amount of extract to the nanoemulsions corresponding to 1.6 mg/mL of total coumarins. We used UFLC to determine that the NAE contained 1.448 mg/mL of total coumarins, and the NSFE contained 1.461 mg/mL. This indicates a successful incorporation of 90.52% and 91.30% relative to the initial value added, respectively.

The nanoemulsions obtained through HPH display droplet sizes ranging from 127 to 162 nm and a viscosity of approximately 1.0 cP. Droplet size plays a fundamental role as it impacts various properties, including viscosity. More spherical drops generally facilitate flowing compared to smaller or distorted droplets, which are prone to coalescence [24]. Uniformity of droplet size distribution is measured according to the PDI, and the nanoemulsions are generally referred to as ‘monodispersed’ if the PDI is lower than 0.2 [25]. According to this parameter, all three formulations are monodispersed, showing a PDI lower than 0.145. In addition, the formulations exhibited a pH above 5.0, which is compatible with the natural skin surface pH [26].

Regarding the ζ-potential, the nanoemulsions displayed negative values due to the presence of negatively charged phospholipids, such as phosphatidylethanolamine, phosphatidylserine, and phosphatidic acid, in the egg lecithin, as described in previous studies [14]. The values (in modulus) ranged from approximately −21 mV to −39 mV, providing good stability to the formulations. A higher ζ-potential was observed when the nanoemulsion was loaded with AE (NAE), suggesting the presence of some compounds at the oil/water interface of this formulation.

### 3.2. Permeation/Retention Assay in Intact and Impaired Skin

To assess the accumulative ability of the coumarins in the tissues in which *S. schenkii* establishes infections, the distribution of coumarins present in the formulations (NAE and NSFE) through intact and impaired porcine skin was evaluated [20]. As can be seen in Table 3, after 8 h of the experiment in intact skin, the major content of total coumarins was concentrated in the dermis, followed by the viable epidermis, for the two formulations (NAE and NSFE), without any significant difference between them. The total coumarin count found in the dermis and viable epidermis for intact skin was 3.14 µg/cm^2^ for NAE and 3.18 µg/cm^2^ for NSFE. A significant increase (*p* < 0.05) in the permeation of total coumarins from NAE and NSFE was detected in the dermis and in the receptor fluid when the stratum corneum was removed. In the impaired skin, the amount of total coumarins in the dermis and epidermis increased to 3.87 µg/cm^2^ and 3.70 µg/cm^2^ for NAE and NSFE, respectively. Removal of the most superficial layer of the skin, the stratum corneum, allowed for a deeper reach of the formulations. These findings are especially important when the target of treatment is to combat subcutaneous fungi, such as *S. schenckii*. As observed in the dermal layer, the amounts of coumarins that permeate to the receptor fluid increase when the skin is impaired, presenting significant differences. In this case, the concentrations of 0.70 µg/cm^2^ (NAE) and 0.59 µg/cm^2^ (NSFE) in intact skin increased to 1.76 and 2.24 µg/cm^2^ in impaired skin, respectively. These results represent threefold and fourfold increases in the permeation profile when the skin is impaired. This behavior occurs due to removal of the upper layer of the skin (stratum corneum), which is intended to provide a physical barrier to protect the skin [26]. Once the skin is impaired, it may allow increased permeation and/or absorption of the compounds. In the case of infections caused by *S. schenckii*, which affect the inner layers of the skin, this study presents interesting treatment alternatives that are able to reach these layers.

### 3.3. Microscopy Analyses

**Figure 3:**
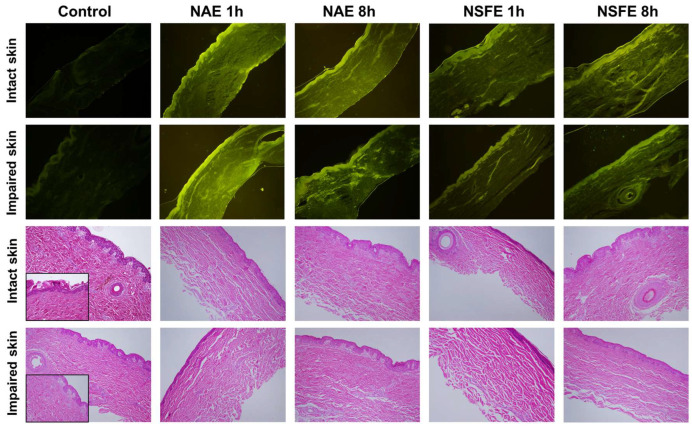
Representative hematoxylin/eosin-stained histological (lower images) and fluorescence (upper images) images of intact and impaired skin, as control, treated with NAE for 1 h and 8 h, and treated with NSFE for 1 h and 8 h. Notes: histological images show no skin damage after treatment with the nanoemulsions. The confocal images revealed that the fluorescence was distributed throughout the skin layers when the dye was incorporated into nanoemulsions. Images were obtained after 1 h and 8 h of permeation/retention studies using a Franz diffusion cell. Images were obtained at 100× and 400× magnifications. NBD-PE was used as fluorescent dye in confocal images. NAE: aqueous extract-loaded nanoemulsion; NSFE: supercritical fluid extract-loaded nanoemulsion.

**Figure 4:**
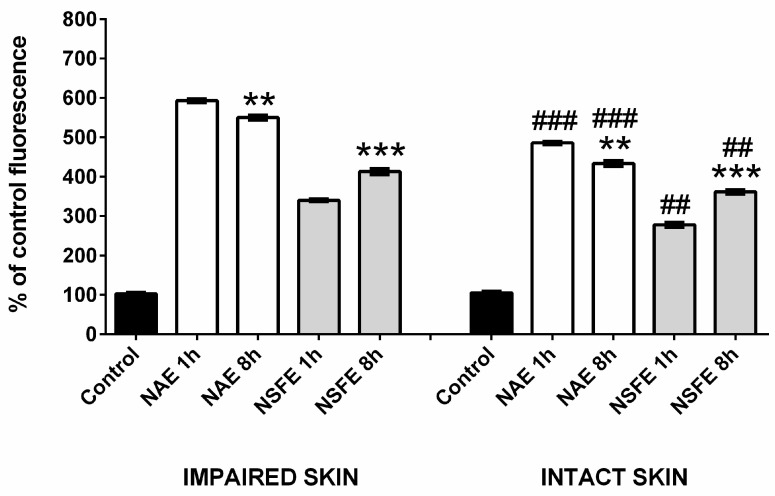
Fluorescent intensity levels detected for impaired or intact skin relative to the percentage of fluorescence emitted by the control tissue. Statistically significant differences were determined through ANOVA with Tukey’s post hoc (* *p* < 0.05, ** *p* < 0.005, *** *p* < 0.0005). Differences between intact and impaired tissues are represented by (#), while differences between 1 h and 8 h of incubation with the same nanoemulsion are represented by (*).

A confocal microscopy evaluation was carried out to shed light on the increased retention in the skin, with histological analyses performed to assess the safety of the treatments. NBD-PE was used as a fluorescence marker as it is easily dispersed in the interface of the nanoemulsions, and the confocal images revealed that the fluorescence was distributed throughout the skin layers. At the top of Figure 3, it is possible to observe the confocal images, and Figure 4 shows the fluorescence intensity levels captured from the images. As can be seen, a significant increase in fluorescence (*p* < 0.001) was detected for impaired skin for both formulations in 1 and 8 h, which corroborates the results concerning coumarin retention achieved in the previous experiment. There is also a difference (*p* < 0.001) between the formulations at 1 and 8 h for both intact and impaired skin. The NAE formulation presented higher fluorescence (approximately 550.3%) than the NSFE formulation (approximately 413%) after 8 h, endorsing the permeation assays in which higher amounts of coumarins were found in the receptor fluid collected from NSFE Franz cells. The tape stripping technique was employed for impairment of the skin, which was subsequently followed by a histological analysis (H&E), which was considered suitable. This analysis enabled the differentiation of the stratum corneum, the viable epidermis, and the dermis in intact skin, as well as the partial removal of the stratum corneum after tape stripping (Figure 3; detailed images of control stratum corneum, 400× magnification) [27]. No signs of skin damage after the treatments were observed in the histological analyses of the samples (Figure 3; lower images).

### 3.4. Antifungal Activity

The probability of identifying antifungal properties in plants is greater when they are known to have ethnopharmacological uses. Considering the use of species of *Pterocaulon* to treat diseases diagnosed as mycosis, Stopiglia et al. investigated different extracts of *Pterocaulon* and demonstrated the antifungal activity against *S. schenckii* [8]. These results indicate that these species are worth further investigating regarding their activity against this fungus. In this sense, this study aimed to evaluate the antifungal activity of *P. balansae* extract-loaded nanoemulsions against some strains of this species. The nanoemulsions were tested against five strains of *S. schenckii*, which included 31 UCS, MLS, Sta Casa 1, STT, and ATCC 201679.

The geometric mean of the MIC values and the MIC ranges of the evaluated sporothricosis agents are presented in Table 4. The results showed an MIC value above 1000 μg/mL for both extracts (AE and SFE) in all of the strains analyzed. In a previous study, values ranging from 312 to 1250 μg/mL were also found for plant extracts [8]. However, those extracts were obtained with organic solvents (hexane, dichloromethane). When incorporated into a lipid release system, MIC values decreased fourfold, with MIC values of 250 µg/mL. This was the lowest antifungal concentration found for this species (*P. balansae*) when treating *S. schenckii* strains. The values of the MIC for itraconazole (positive control) for *S. schenckii* were 2.0 (31 UCS), 2.0 (MLS), >16.0 (Sta Casa 1), 2.0 (ATCC 201679), and >16.0 (STT). The markedly pronounced activity of the nanoemulsions when compared to the extracts may be linked to the composition of these formulations, which comprise phospholipids that may interact and destabilize the cellular wall of these fungi, provoking cell death [14,28].

## 4. Conclusions

This study showed the feasibility of incorporating aqueous and supercritical fluid *P. balansae* extracts in nanoemulsions, which demonstrated satisfactory physicochemical properties. These formulations were capable of releasing coumarin content through impaired and intact skin, with a larger amount of coumarins detected in the receptor fluid and the dermis, reaching the inner layers of the skin. Consequently, the formulations demonstrated an important antifungal activity against *S. schenckii* strains, and more studies may be conducted because this is an interesting alternative treatment for widespread sporotrichosis infections.

## Figures and Tables

**Table 1:** Composition of nanoemulsions.

Composition	NB	NAE	NSFE
Medium chain triglycerides (MCT) (%)	16.0	16.0	16.0
Egg lecithin (%)	4.0	4.0	4.0
Polysorbate 80 (%)	1.0	1.0	1.0
AE (mg/mL)	-	1.60	-
SFE (mg/mL)	-	-	1.60
Water to	100.0	100.0	100.0

NB: blank nanoemulsions; NAE: aqueous extract-loaded nanoemulsion; NSFE: supercritical fluid extract-loaded nanoemulsion. Content of coumarins adjusted by yield (AE: 9.5% and SFE: 42.26%).

**Table 2:** Physicochemical properties of nanoemulsions.

Parameter	NB	NAE	NSFE
Size (nm)	133.02 (3.87) ^a^	162.02 (6.59) ^ac^	127.49 (4.18) ^c^
PDI	0.145 (0.02)	0.111 (0.02)	0.096 (0.03)
ζ-potential (mV)	−38.92 (1.77) ^b^	−32.62 (6.03)	−21.20 (3.42) ^b^
pH	4.69 (0.03) ^ab^	5.33 (0.01) ^ac^	4.46 (0.08) ^bc^
Viscosity (cP)	0.96 (0.007) ^ab^	1.08 (0.011) ^a^	1.06 (0.005) ^b^
Total coumarin content (mg/mL)	-	1.448 (0.04)	1.461 (0.06)

PDI: Polydispersity index; NB: blank nanoemulsion; NAE: aqueous extract-loaded nanoemulsion; NSFE: supercritical fluid extract-loaded nanoemulsion. (Mean and SD for three determinations, where SD is standard deviation). ^a^ Significantly different (*p* < 0.05) for NB and NAE. ^b^ Significantly different (*p* < 0.05) for NB and NSFE. ^c^ Significantly different (*p* < 0.05) for NSFE and NAE.

**Table 3:** Distribution profile of total coumarins (TC) from NAE and NSFE in impaired and intact porcine ear skin layers after 8 h of permeation/retention study.

		NAE Mean (SD)	NSFE Mean (SD)
		µg/cm^2^	µg/cm^2^
Intact skin	*Stratum corneum*	0.86 (0.23)	0.52 (0.06)
	*Viable epidermis*	1.16 (0.40)	1.03 (0.13)
	*Dermis*	1.98 (0.39) ^a^	2.15 (0.14) ^c^
	*Receptor fluid*	0.70 (0.11) ^b^	0.59 (0.05) ^d^
Impaired skin	*Epidermis*	1.02 (0.74)	0.91 (0.16)
	*Dermis*	2.85 (0.81) ^a^	2.79 (0.42) ^c^
	*Receptor fluid*	1.76 (0.51) ^b^	2.24 (0.23) ^d^

Statistically significant difference between impaired and intact skin (*p* < 0.05). ^a,b^ for NAE and ^c,d^ for NSFE. NAE: aqueous extract-loaded nanoemulsion; NSFE: supercritical fluid extract-loaded nanoemulsion. (Mean and SD for three determinations, where SD is standard deviation); ANOVA and Tukey’s post hoc.

**Table 4:** Antifungal activity (MICs in µg/mL) of *P. balansae* extracts and nanoemulsions.

Samples	*Sporothrix schenkii*				
	Ss 31 UCS	Ss MLS	Ss Sta Casa 1	Ss ATCC 201679	Ss STT
AE	>1000	>1000	>1000	>1000	>1000
SFE	>1000	>1000	>1000	>1000	>1000
NAE	250	250	250	250	250
NSFE	250	250	250	250	250

Notes: AE: aqueous extract; SFE: supercritical fluid extract; NAE: aqueous extract-loaded nanoemulsion; NSFE: supercritical fluid extract-loaded nanoemulsion.

## Data Availability

There are no new data associated with this article.
